# Original research: longitudinal evaluation of cognitively demanding daily function using performance-based functional assessment highlights heterogeneous trajectories in cognitive and functional abilities in people with Parkinson’s disease

**DOI:** 10.3389/fnins.2023.1200347

**Published:** 2023-06-26

**Authors:** Tara C. Carlisle, Angela J. Fought, Kaitlin E. Olson, Natalie Lopez-Esquibel, Abigail Simpson, Luis D. Medina, Samantha K. Holden

**Affiliations:** ^1^Department of Neurology, University of Colorado School of Medicine, Aurora, CO, United States; ^2^Behavioral Neurology Section, Department of Neurology, University of Colorado School of Medicine, Aurora, CO, United States; ^3^University of Colorado Movement Disorders Center, Aurora, CO, United States; ^4^University of Colorado Alzheimer’s and Cognition Center, Aurora, CO, United States; ^5^Department of Biostatistics and Informatics, Colorado School of Public Health, Aurora, CO, United States; ^6^Department of Psychology, University of Houston, Houston, TX, United States; ^7^Movement Disorders Section, Department of Neurology, University of Colorado School of Medicine, Aurora, CO, United States

**Keywords:** Parkinson’s disease, activities of daily living, cognitive dysfunction, dementia, functional assessment

## Abstract

**Background:**

Longitudinal assessment of functional abilities in Parkinson’s disease (PD) is needed to determine the efficacy of cognitive interventions in providing meaningful improvements in daily life. Additionally, subtle changes in instrumental activities of daily living may precede a clinical diagnosis of dementia and could aid earlier detection of and intervention for cognitive decline.

**Objective:**

The primary goal was to validate the longitudinal application of the University of California San Diego Performance-Based Skills Assessment (UPSA). An exploratory secondary goal was to determine whether UPSA may identify individuals at higher risk of cognitive decline in PD.

**Methods:**

Seventy participants with PD completed the UPSA with at least one follow-up visit. Linear mixed effects modeling was used to identify associations between baseline UPSA score and cognitive composite score (CCS) over time. Descriptive analysis of four heterogeneous cognitive and functional trajectory groups and individual case examples was performed.

**Results:**

Baseline UPSA score predicted CCS at each timepoint for functionally impaired and unimpaired groups (*p* < 0.01) but did not predict the rate change in CCS over time (*p* = 0.83). Participants displayed heterogenous trajectories in both UPSA and CCS during the follow-up period. Most participants maintained both cognitive and functional performance (*n* = 54), though some displayed cognitive and functional decline (*n* = 4), cognitive decline with functional maintenance (*n* = 4), and functional decline with cognitive maintenance (*n* = 8).

**Conclusion:**

The UPSA is a valid measure of cognitive functional abilities over time in PD. Given the heterogeneity of functional and cognitive trajectories, this performance-based assessment did not predict cognitive decline with this relatively short follow-up. Further work is needed to understand longitudinal functional assessments in PD-associated cognitive impairment.

## 1. Introduction

Guidance from the U.S. Food and Drug Administration recommends incorporating functional assessments in Alzheimer’s disease clinical trials, with the goal of ensuring meaningful improvements in daily life with any potential interventions for cognitive impairment (Food and Drug Administration (FDA), 2018). This requires an understanding of how performance-based functional assessments (PBFAs) measure change over time (Lassen-Greene et al., 2017) and methods to avoid practice effects with repeated assessments (Bell et al., 2021). There has been a move towards validating longitudinal PBFAs in Alzheimer’s disease research (Lassen-Greene et al., 2017; Goldberg et al., 2020), but there is only one reported study using a longitudinal PBFA in individuals with Parkinson’s disease (PD; Beyle et al., 2018). This is despite cognitive changes being present in 10–20% of individuals newly diagnosed with PD (Aarsland et al., 2009; Weintraub et al., 2015). There are high rates of conversion to dementia in people with PD when followed longitudinally (Aarsland et al., 2003), with approximately 80% developing dementia during the course of PD (Hely et al., 2008). As there are currently no disease-modifying therapies for PD or its associated cognitive impairment, it is important to develop and validate functional assessments for PD-specific longitudinal clinical trials.

Additionally, given heterogenous cognitive trajectories in PD, there have been multiple attempts to predict those with PD at the highest risk of developing cognitive decline reliably and early (Anang et al., 2014, 2017; Schrag et al., 2017; Dawson et al., 2018). Deficits in activities of daily living (ADLs) can be present prior to global cognitive impairment and contribute to incident dementia risk (Tomaszewski Farias et al., 2018; Feger et al., 2020; Barthold et al., 2021; Dorociak et al., 2021; Lindbergh et al., 2016). In mild cognitive impairment (MCI), there is increasing evidence of functional deficits in instrumental ADLs (iADLs) prior to a clinical diagnosis of dementia (Pérès et al., 2008; Fauth et al., 2013; Jekel et al., 2015), including in people with PD (Rosenthal et al., 2010; Pirogovsky et al., 2014). Informant-based and self-reported scales of functional impairment are associated with conversion from MCI to dementia (Luck et al., 2012; Devanand et al., 2017; Cloutier et al., 2021).

Given that subtle iADL impairment may precede a clinical diagnosis of dementia, formal evaluation of functional abilities by self-report, caregiver report, PBFA, and direct behavioral observation have become increasingly prevalent in dementia prediction research. However, iADLs are not reliant on cognition alone, with physical, environmental, and educational factors also contributing (Bruderer-Hofstetter et al., 2020). Therefore, understanding the complexity of iADLs in the real world and how impairment may predict later dementia requires a multifaceted approach. For example, our previous work demonstrated that performance on the University of California San Diego Performance-Based Skills Assessment (UPSA) score is weakly correlated with motor severity but strongly associated with global cognitive score in people with PD; therefore, the UPSA may serve as a combined cognitive and motoric outcome measure for PD (Holden et al., 2018).

Multiple questionnaire-and interview-based assessments have been used to assess for changes in iADLs in PD, e.g., Clinical Dementia Rating Scale Sum of Boxes (CDR-SOB) (Gallagher et al., 2021), Penn Parkinson’s Daily Activities Questionnaire-15 (PDAQ-15; Cholerton et al., 2020), however these approaches may not be sensitive to subtle and early stages of functional decline. Multiple PBFAs have been utilized in PD research in addition to UPSA (Holden et al., 2018; Table 1) (Giovannetti et al., 2012; Manning et al., 2012; Pirogovsky et al., 2012, 2013, 2014; Higginson et al., 2013; Martin et al., 2013; Foster, 2014; Lanni et al., 2014; Glonnegger et al., 2016; Lopez et al., 2019; de Oliveira et al., 2020; Sulzer et al., 2020; Foster and Doty, 2021). Many of these studies provide evidence that non-demented participants with PD already display iADL impairment based on PBFA testing (Manning et al., 2012; Martin et al., 2013; Foster, 2014; Lanni et al., 2014; Lopez et al., 2019; de Oliveira et al., 2020; Sulzer et al., 2020; Foster and Doty, 2021). Questionnaires appear to be weakly associated with PBFAs (Deck et al., 2019; de Oliveira et al., 2020), suggesting that questionnaire-based functional assessments and PBFAs may provide unique clinical information. PBFAs are objective measures based on direct observation of common daily tasks, and therefore less subject to bias from degree of insight, recall, familiarity with daily routine, or caregiver burden. In PD specifically, PBFAs can also allow for evaluation of relative contributions of cognitive and motoric impairments to iADL performance, both cross-sectionally and longitudinally. The primary goal of the current study was to perform a longitudinal validation of the UPSA in PD to determine whether UPSA performance continues to track with global cognition over time. A secondary goal was to determine whether baseline UPSA performance may help predict future cognitive decline in people with PD, given evidence that subtle iADL impairments may predate capturing impairments captured by neuropsychological testing. This is an initial analysis of our longitudinal data, while awaiting more time for follow-up of our cohort and potential cognitive changes in participants.

**Table 1:** Performance-based functional assessments used in populations with Parkinson’s disease.

Performance-based functional assessment	Parkinson’s disease references
UCSD performance-based skills assessment	Holden et al. (2018) This study^*^
Shortened direct assessment of functional status	Giovannetti et al. (2012)
Hopkins medication schedule with expanded PD-specific regimen	Manning et al. (2012)
Medication management ability assessment with the advanced finances test	Pirogovsky et al. (2012)
Medication management ability assessment with UPSA finances subdomain	Pirogovsky et al. (2013, 2014)
Financial capacity instrument	Martin et al. (2013)
Timed instrumental activities of daily living	Lanni et al. (2014) Higginson et al. (2013)
Performance assessment of self-care skills	Foster (2014) Foster and Doty (2021)
Multiple object test	Glonnegger et al. (2016) Beyle et al. (2018) *
Revised-observed tasks of daily living	Lopez et al. (2019)
Direct assessment of functional abilities	de Oliveira et al., (2020)
Erlangen test of activities of daily living in mild dementia and MCI	Sulzer et al. (2020)
Erlangen test of activities of daily living	Sulzer et al. (2020)

* Longitudinal assessment of performance-based assessment.

UPSA: UCSD performance-based skills assessment

## 2. Methods

### 2.1. Participants

One hundred two participants with idiopathic PD without dementia were recruited from the University of Colorado (Aurora, CO) Movement Disorders Center and community movement disorders specialists to participate in this study between January 14, 2016 and June 18, 2021. All participants were offered the opportunity to participate in longitudinal assessments after baseline visits. If a participant converted to PD dementia (PDD) at a follow-up visit, they were subsequently removed from the future follow-up pool as they met the defined study end-point of conversion to dementia. A total of 70 participants had baseline and at least one follow-up assessment during the study period. Follow-up duration from baseline was defined as follows: year 1 as 12 ± 6 months, year 2 as 24 ± 6 months, and year 3 as 36 ± 6 months. Repeat assessments were performed no sooner than 6-month intervals to avoid practice effects.

Inclusion criteria included diagnosis of PD by Queen’s Square Brain Bank criteria (Hughes et al., 1992), age 40–90 years, and English as a primary language. Exclusion criteria included active or severe depression or anxiety defined as a Hospital Anxiety and Depression Scale (HADS)-Depression (HADS-D) or HADS-Anxiety (HADS-A) subscore > 11, atypical or secondary parkinsonism, and other comorbid neurologic conditions (i.e., multiple sclerosis, epilepsy, traumatic brain injury). Participant demographic information, educational and occupational history, current medications, and medical history were collected. The study was approved by the Colorado Multiple Institutional Review Board (protocol #15-0170) and all participants provided written informed consent.

### 2.2. Data handling

Study data were collected and managed using Research Electronic Data Capture (REDCap) tool hosted at the University of Colorado as part of the Colorado Clinical and Translational Sciences Institute Development and Informatics Service Center (Harris et al., 2009, 2019).

### 2.3. Administered assessments

#### 2.3.1. Clinical scales

Collected clinical scales were previously reported in our UPSA validation study (Holden et al., 2018), including levodopa equivalent daily dose (LEDD) (Tomlinson et al., 2010), clinical interview to assess for current functional impairments (performed by a neurologist experienced in dementia evaluations [SH]) guided by the Lawton iADL scale (Lawton and Brody, 1969), Mattis Dementia Rating Scale (DRS-2) as a measure of global cognition (Schmidt et al., 2005) recommended for use in PD by the International Parkinson and Movement Disorder Society (MDS) Rating Scales Review Committee (Skorvanek et al., 2018), motor examination in the “ON” PD medication state (performed by a movement disorders neurologist [SH]) with Unified Parkinson Disease Rating Scale (UPDRS) Part III (Martinez-Martin et al., 1994), and disease staging with the Hoehn and Yahr (H&Y) scale (Hoehn and Yahr, 1967). Additional scales include the clinician-performed Apathy Evaluation Scale (AES-C) (Marin et al., 1991), HADS (Zigmond and Snaith, 1983), PDAQ-15 (Brennan et al., 2016), Parkinson’s disease-Cognitive Functional Rating Scale (PD-CFRS) (Kulisevsky et al., 2013), and Parkinson’s Disease Questionnaire (PDQ-39) (Jenkinson et al., 1997).

#### 2.3.2. Functional assessment

The UPSA includes assessments of five functional subdomains (e.g., Financial, Communication, Planning/Organization, Travel, and Household Management) as previously described (Patterson et al., 2001) and validated in PD (Holden et al., 2018). Participants receive a score in each subdomain (range 0–20) that are summed to determine a total score (range 0–100). The UPSA evaluator was blinded to the cognitive classification and neuropsychological testing scores of the participants at the time of UPSA administration.

#### 2.3.3. Neuropsychological battery

A neuropsychological battery comprised of ten tests with two in each of the following five cognitive domains was administered: (1) Executive Function: Trail Making Test-B (Reitan and Wolfson, 1993) and Controlled Oral Word Association Test (Benton and Hamsher, 1989); (2) Memory: California Verbal Learning Test-II (Delis et al., 2000) and Visual Reproduction recall and recognition from the Wechsler Memory Scale-Revised (Wechsler, 1987); (3) Language: Boston Naming Test (Kaplan et al., 1983) and Category Fluency: Animal Naming (Morris et al., 1989); (4) Attention: Brief Test of Attention (Schretlen et al., 1996) and Symbol Digit Modalities Test (Smith, 1973); and (5) Visuospatial Function: Judgment of Line Orientation (Benton et al., 1978) and Intersecting Pentagons (Folstein et al., 1983). This battery was chosen based on input from neuropsychologists experienced in performing cognitive assessments in PD (Holden et al., 2018) and from previous work validating the MDS Task Force diagnostic criteria for PD-MCI (Goldman et al., 2015). Raw scores were converted to z-scores based on normative data for each of the individual neuropsychological tests, drawn from either testing manuals (Smith, 1973; Benton et al., 1978; Wechsler, 1987; Reitan and Wolfson, 1993; Delis et al., 2000; Schmidt et al., 2005) or additional normative studies (Drane et al., 2002; Ruff and Parker, 1993; Tombaugh and Hubley, 1997; Tombaugh et al., 1999). *Z*-scores of each neuropsychological test were averaged into a cognitive composite score used as the outcome measure for global cognition. Cognitive composite scores (CCS) are routine outcome measures in AD research (Langbaum et al., 2014; Burnham et al., 2015) and also utilized in PD cognitive research (Wood et al., 2021). The Montreal Cognitive Assessment (MoCA) was also collected as a global measure of cognition (Nasreddine et al., 2005) but was not included in the CCS.

### 2.4. Cognitive classification

Cognitive classifications were determined at consensus conference attended by at least one neurologist (SH) and one neuropsychologist (LM), both with experience in PD cognition. Other consensus conference participants included additional neurologists, neuropsychologists, and study coordinators. All members of the consensus conference were blinded to UPSA scores. Possible cognitive classifications included normal cognition (PD-NC), mild cognitive impairment (PD-MCI), or dementia (PDD) based on MDS Task Force diagnostic guidelines (Emre et al., 2007; Litvan et al., 2012). Impairment on an individual neuropsychological test was defined as a z-score > 1.5 standard deviations (SDs) below normative data (Goldman et al., 2013). PD-NC was defined as all z-scores ≤1.5 SDs below normative data. Classification of cognitive impairment (PD-MCI, PDD) required impairment on two neuropsychological tests in one cognitive domain (PD-MCI) or one neuropsychological test in two different cognitive domains (PD-MCI, PDD). Designation of PDD was based on significant functional impairment due to cognitive symptoms per clinical interview (SH). Conversion to more impaired cognitive class at each follow-up was based on comparison between the current and most recent prior cognitive classification. Participants that converted to PDD in their final follow-up were included in the analysis.

### 2.5. Motor classification

Motor group classification was performed as previously described and modified (Stebbins et al., 2013; Wojtala et al., 2019). In brief, tremor (i.e., UPDRS Part III items 20 [resting tremor] and 21 [postural tremor of hands] divided by number of subitems [7]) and non-tremor (i.e., UPDRS Part III items 18 [speech], 19 [face expressions], 22 [rigidity], 27 [arising from chair], 28 [posture], 29 [gait], 30 [postural instability], and 31 [bradykinesia/hypokinesia of the body] divided by the number of subitems [12]) scores were calculated. Tremor dominant (TR-D) was classified as tremor/non-tremor score ratio ≥ 1.5 or a positive tremor score and a zero non-tremor score. Postural instability and gait difficulty dominant (PIGD-D) was classified as tremor/non-tremor score ratio ≤ 1.0 or a zero tremor score and a positive non-tremor score. Intermediate ‘not determined’ (ND) was classified as > 1 to ≤ 1.5 or zero scores for both tremor and non-tremor scores. Presence of postural instability was defined as a non-zero postural instability score (i.e., UPDRS Part III subitem 30) (Urso et al., 2021).

### 2.6. Statistical analysis

Many of the variables displayed skew and therefore the median and interquartile range (IQR; Q1, Q3) were used to represent the data descriptively. There were very few missing data with only one missing CCS value and three missing LEDD values.

The cohort was divided based on baseline UPSA total scores into unimpaired (≥ 85, *n* = 45) and impaired (<85, *n* = 57). This conservative cut-off score was chosen based on discriminant validity analysis (PD-NC versus PD-MCI discrimination) from our previous validation study allowing for detection of even subtle iADL impairments at early stages (Holden et al., 2018). Linear mixed effects modeling was used to identify the associations between baseline UPSA total score and CCS, time (years) including up to 3-year follow-up, and the interaction between time and CCS. A random intercept was used to account for the correlation between different visits for the same participant. In addition to normal model output, estimated means and their standard deviations were calculated to understand the relationship of the interaction term and the outcome.

### 2.7. Heterogenous trajectory stratification

To better understand the heterogeneous longitudinal trajectories of UPSA total score and CCS over time, we defined decline as >0.5 SD worsening in CCS and/or UPSA (equivalent to > 6.4 point decline, or 0.5 SD from mean UPSA score, for participants without dementia in the validation study) from baseline to follow-up including to year 5. A conservative cut-off of > 0.5 SD for meaningful cognitive decline was chosen for this highly educated cohort to capture even subtle changes over the relatively short follow-up. This produced four distinct categories: (1) decline in both UPSA total score and CCS (i.e., cognitive and functional decliner, or double decliner [DD]), (2) decline in CCS alone (i.e., cognitive decliner, functional maintainer [CDFM]), (3) decline in UPSA total score alone (i.e., functional decliner, cognitive maintainer [FDCM]), or (4) no decline in UPSA or CCS (i.e., cognitive and functional maintainer, or double maintainer [DM]). Statistical comparisons were not possible due to the small sample sizes; however, absolute proportion comparisons were performed in this descriptive approach.

### 2.8. Case vignettes

To further describe the variable cognitive and functional trajectories, single cases for each of the four categories were explored in further detail as case vignettes. All selected cases had PD-NC cognitive classification at baseline. Cases were additionally chosen to maximize the duration of follow-up. Again, absolute comparisons were performed in this exploratory approach.

## 3. Results

### 3.1. Clinical characteristics

Table 2 displays the demographic and clinical features of our PD cohort from baseline (n = 102) to longitudinal follow-up including years 1 (*n* = 43), 2 (*n* = 31), and 3 (*n* = 13). There was no statistical difference in baseline age, sex, years of education, disease duration, UPDRS Part III, LEDD, UPSA total score, or cognitive class between those baseline participants who returned for follow-up visits and those who did not; however, the group that opted for follow-up had significantly higher baseline MoCA score (*p* = 0.018) and CCS (*p* = 0.027).

**Table 2:** Participant demographic and clinical features at yearly assessments.

	Baseline			Year 1 (*n* = 43)	Year 2 (*n* = 31)	Year 3 (*n* = 13)
	All	With follow-up	Without follow-up			
Age (years)	68.0 (63.2, 74.0)	68.0 (63.0, 73.8)	70.5 (65.0, 74.0)	72.0 (64.5, 75.0)	70.0 (65.0, 76.0)	68.0 (67.0, 71.0)
Sex (% female)	33 (32.4%)	45 (64.29%)	24 (75.0%)	14 (32.6%)	11 (35.5%)	6 (46.2%)
Education (years)	17.0 (15.2, 18.0)	17.0 (16.0, 18.0)	16.5 (14.0, 18.0)	18.0 (16.0, 18.5)	17.0 (15.5, 18.0)	16.0 (16.0, 18.0)
PD Duration (years)	3.0 (2.0, 6.0)	3.0 (2.0, 6.0)	3.0 (2.0, 6.3)	4.0 (3.0, 8.5)	5.0 (3.8, 10.0)	5.0 (5.0, 7.0)
UPDRS Part III	27.0 (19.2, 35.0)	27.0 (19.0, 31.8)	29.5 (20.0, 38.3)	30.0 (23.8, 37.5)	25.5 (20.0, 34.0)	30.0 (21.0, 35.0)
LEDD (mg/day)	400.0 (200.0, 720.0)	440.0 (225.0, 750.0)	340.0 (150.0, 609.5)	600.0 (400.0, 833.8)	600.0 (350.0, 1004.0)	595.0 (520.0, 869.0)
UPSA total	83.0 (77.0, 89.0)	84.0 (79.0, 89.8)	80.5 (73.0, 87.0)	84.0 (78.5, 90.5)	85.0 (81.0, 90.0)	84.0 (77.0, 94.0)
MoCA	27.0 (25.0, 29.0)	28.0 (25.0, 29.0)	26.0 (21.0, 28.0)	28.0 (24.0, 29.0)	27.0 (24.0, 29.0)	27.0 (25.0, 28.0)
PD-NC (%)	75 (73.5%)	55 (78.6%)	20 (62.5%)	28 (65.1%)	26 (86.7%)	12 (92.3%)
PD-MCI (%)	27 (26.5%)	15 (21.4%)	12 (37.5%)	8 (18.6%)	1 (3.3%)	1 (7.7%)
PDD (%)	N/A	N/A	N/A	7 (16.3%)	3 (10.0%)	0 (0.0%)
Conversion to more impaired cognitive class	N/A	N/A	N/A	*n* = 11	*n* = 5	*n* = 1

Data presented as median (Q1, Q3) unless otherwise noted.

LEDD: levodopa equivalent daily dosing; MoCA: Montreal Cognitive Assessment; PD: Parkinson’s disease; PDD: Parkinson’s disease dementia; PD-MCI: Parkinson’s disease mild cognitive impairment; PD-NC: Parkinson’s disease normal cognition; UPDRS: Unified Parkinson Disease Rating Scale; UPSA: UCSD Performance-Based Skills Assessment.

Table 3 displays the baseline demographic and clinical features of our PD cohort when divided into baseline unimpaired (≥ 85, *n* = 45) and impaired (<85, *n* = 57) UPSA total score. At baseline, participants with impaired UPSA were significantly older (*p* < 0.001) and less likely to be female (*p* = 0.023) with significantly lower CCS (*p* < 0.001) and higher UPDRS Part III motor scores (*p* < 0.001) compared to participants with unimpaired UPSA. The impaired baseline UPSA group had 26.2% (*n* = 22) PD-MCI compared to only 11.1% (*n* = 5) in the unimpaired baseline UPSA group. There was no statistically significant difference in disease duration (*p* = 0.067) or years of education (*p* = 0.931) in this highly educated cohort between baseline impaired and unimpaired groups.

**Table 3:** Participant demographic and clinical features by baseline UPSA performance group.

	Impaired baseline UPSA (Total Score < 85, *n* = 57)	Normal baseline UPSA (Total Score ≥ 85, *n* = 45)	*p*-Value
Age (years)	71.0 (65.0, 74.0)	66.0 (61.0, 71.0)	<0.001
Sex (% female)	13 (22.8%)	20 (44.4%)	0.023
Education (years)	18.0 (14.0, 18.0)	17.0 (16.0, 18.0)	0.931
PD Duration (years)	3.0 (2.0, 7.0)	3.0 (1.5, 5.0)	0.067
UPDRS Part III	30.0 (23.0, 36.0)	23.0 (17.0, 34.0)	<0.001
CCS	−0.1 (−0.3, 0.3)	0.4 (0.0, 0.7)	<0.001
Cognitive class	PD-NC: 35 (61.4%) PD-MCI: 22 (38.6%)	PD-NC: 40 (88.9%) PD-MCI: 5 (11.1%)	N/A

Data presented as median (Q1, Q3) unless otherwise noted.

CCS: cognitive composite score; PD: Parkinson’s disease; PDD: Parkinson’s disease dementia; PD-MCI: Parkinson’s disease mild cognitive impairment; PD-NC: Parkinson’s disease normal cognition; UPDRS: Unified Parkinson Disease Rating Scale; UPSA: UCSD Performance-Based Skills Assessment.

### 3.2. Ceiling/floor effects

We previously reported the psychometric properties of the UPSA in PD, including absence of floor or ceiling effects (Holden et al., 2018). There were no significant floor or ceiling effects with longitudinal UPSA total scores ranging 51–98 (median 83.0; IQR 77.0, 89.0), 46–96 (median 84.0; IQR 78.0, 90.2), 66–95 (median 85.0; IQR 81.0, 90.0), and 73–95 (median 84.0; IQR 77.0, 94.0) for baseline, year 1, year 2, and year 3, respectively.

### 3.3. Validity of the UPSA over time

We previously showed that UPSA total score correlates with global cognition (DRS-2) cross-sectionally across a spectrum of cognitive abilities in PD (Holden et al., 2018). Similarly for this longitudinal study, baseline UPSA total score significantly correlates with baseline CCS (*r* = 0.657, *p* < 0.0001) when adjusted for age, level of education, PD disease duration, and LEDD. This significant correlation between UPSA and CCS is maintained at year 1 (*r* = 0.599, *p* = 0.0001) and year 2 (*r* = 0.391, *p* = 0.044) follow-ups, but is not present for the year 3 follow-up (*r* = 0.007, *p* = 0.98).

**Figure 1:**
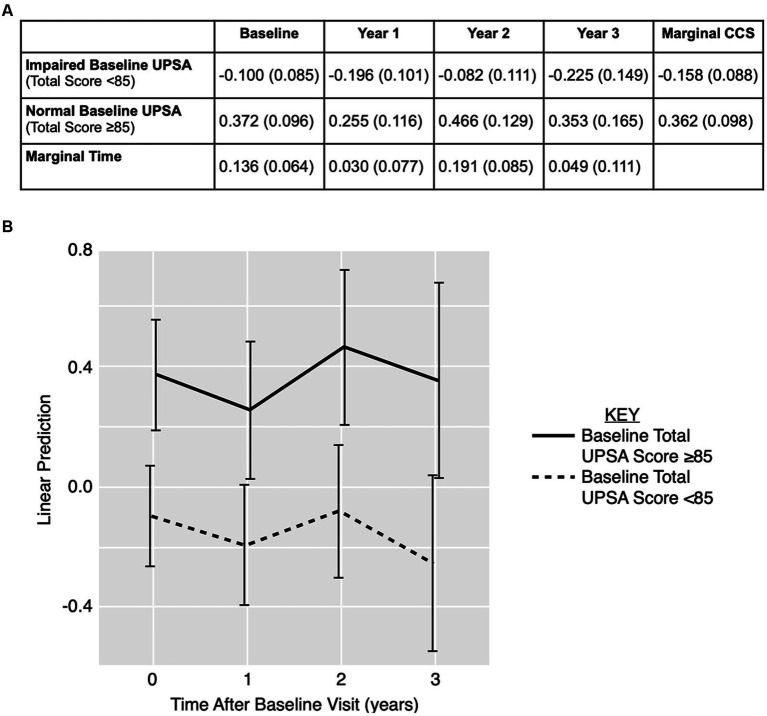
Linear prediction model for cognition over time using UPSA total score over time. **(A)** CCS data displayed as estimated means (standard error) stratified by baseline UPSA total score and visit year. **(B)** Estimated means and 95% confidence interval for CCS outcome for baseline impaired (< 85; dotted line) and unimpaired (≥ 85; solid line) UPSA. CCS: cognitive composite score; UPSA: UCSD Performance-Based Skills Assessment.

Figure 1A displays CCS stratified by baseline UPSA total score and visit year. The time variable did not significantly predict CCS (*p* = 0.17). The baseline UPSA total score significantly predicted CCS at each timepoint for both groups (i.e., impaired baseline UPSA, unimpaired baseline UPSA) in the model (*p* < 0.01), but baseline UPSA did not predict the rate of change in CCS over time (*p* = 0.83).

Figure 1B displays the linear prediction model using baseline UPSA total score to predict CCS over time with 95% confidence intervals. Although there is little separation in the model between impaired and unimpaired baseline UPSA total scores in predicting CCS over time, there is a trend towards lower CCS in the impaired baseline UPSA total score group.

### 3.4. Heterogeneity of longitudinal UPSA total scores and cognitive trajectories

**Figure 2:**
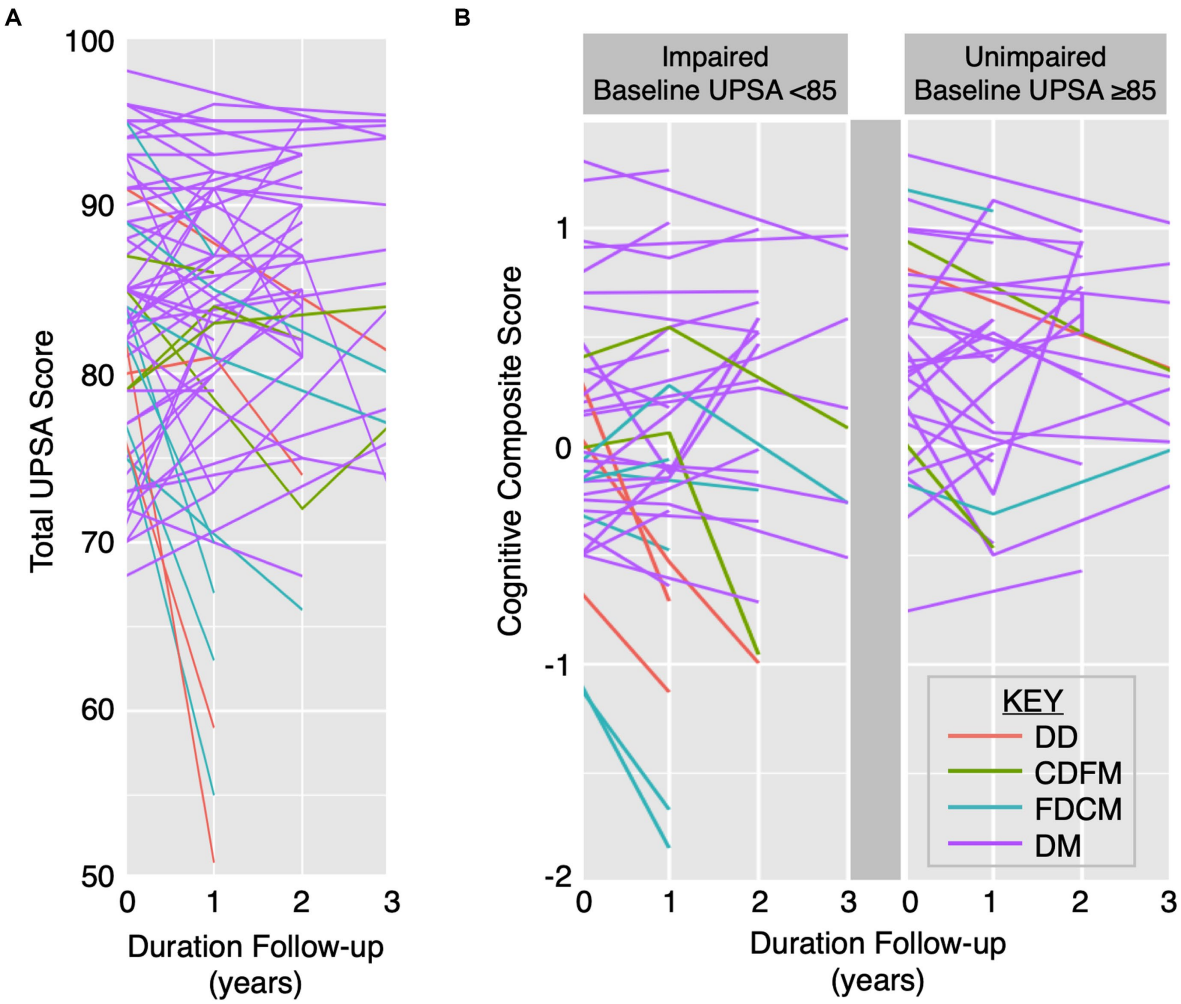
Heterogenous longitudinal UPSA total score and CCS trajectories. Trajectories of UPSA total score **(A)** and CCS by impaired baseline UPSA versus unimpaired baseline UPSA **(B)** over time by specific cognitive and functional category: (1) DD (red), (2) CDFM (green), (3) FDCM (cyan), and (4) DM (purple). CDFM: cognitive decliner, functional maintainer; DD: double decliner (i.e., cognitive and functional decliner); DM: double maintainer (i.e., cognitive and functional maintainer); FDCM: functional decliner, cognitive maintainer.

Participant UPSA total scores and CCS displayed heterogenous trajectories over time (Figure 2). Stratifying based on decline or maintenance in UPSA and CCS, there were 4 DD, 4 CDFM, 8 FDCM, and 54 DM participants. Participants with a decline in UPSA score can have either maintained cognitive performance (e.g., FDCM) or decline in cognitive performance (e.g., DD; Figure 2A), highlighting variable changes in cognitive and functional capacities. Each of the four cognitive and functional trajectory categories are present in both the impaired (total score < 85) and unimpaired (total score ≥ 85) baseline UPSA groups (Figure 2B).

Table 4 displays demographic and clinical features of our PD cohort at baseline delineated by the variable longitudinal cognitive and functional trajectories. The groups with functional maintenance (CDFM/DM) were younger than the functional decliner groups (DD/FDCM). The DM group had a higher proportion of females compared to the other longitudinal trajectory groups. The DD group had longer PD duration.

**Table 4:** Baseline demographic and clinical features by cognitive/functional trajectory group.

	Cognitive and functional decliner (DD) (*n* = 4)	Cognitive decliner, functional maintainer (CDFM) (*n* = 4)	Functional decliner, cognitive maintainer (FDCM) (*n* = 8)	Cognitive and functional maintainer (DM) (*n* = 54)
*Demographic characteristics*				
Age (years)	74.5 (70.0, 78.0)	66.5 (61.5, 72.5)	72.5 (71.0, 76.0)	67.0 (62.0, 71.0)
PD duration (years)	8.0 (4.0, 12.5)	4.0 (2.0, 6.5)	4.5 (1.5, 6.0)	3.0 (2.0, 5.8)
Sex (% female)	0.0%	25.0%	25.0%	40.7%
Education (years)	17.5 (16.5, 19.5)	18.5 (16.0, 21.0)	18.0 (16.0, 18.0)	16.5 (16.0, 18.0)
*Disease characteristics*				
UPDRS Part III	36.5 (28.5, 40.0)	26.0 (17.0, 31.0)	31.0 (16.0, 35.0)	25.5 (19.0, 31.0)
Motor phenotype	TR-D: 0 (0.0%) PIGD-D: 3 (75.0%) ND: 1 (25.0%)	TR-D: 0 (0.0%) PIGD-D: 4 (100.0%) ND: 0 (0.0%)	TR-D: 0 (0.0%) PIGD-D: 7 (87.5%) ND: 1 (12.5%)	TR-D: 2 (3.7%) PIGD-D: 44 (81.5%) ND: 8 (14.8%)
Postural instability	4 (100.0%)	1 (25.0%)	5 (62.5%)	20 (37.0%)
LEDD (mg/day)	675 (350, 1,120)	1,065 (875, 1,215)	525 (416, 908)	400 (205.0, 587.5)
PDQ-39	16.4 (12.6, 18.4)	14.0 (9.7, 18.2)	17.2 (5.9, 24.8)	12.6 (6.1, 20.6)
*Mood characteristics*				
HADS-A	2.5 (2.0, 3.5)	3.5 (1.5, 5.5)	2.5 (0.0, 6.0)	1.5 (0.0, 4.0)
HADS-D	2.0 (1.5, 3.5)	2.0 (2.0, 3.0)	1.5 (1.0, 3.0)	1.5 (1.0, 3.0)
AES-C	29.5 (27.0, 34.0)	22.5 (21.0, 26.5)	22.5 (20.0, 24.5)	21.0 (19.0, 26.0)
*Cognitive functioning*				
MoCA	26.0 (23.5, 28.5)	27.0 (24.5, 29.5)	25.5 (23.0, 28.0)	28.0 (26.0, 29.0)
DRS-2	138.0 (136.5, 140.0)	140.5 (139.5, 142.0)	138.5 (137.0, 141.0)	141.0 (139.0, 142.0)
Cognitive Composite	0.18 (−0.31, 0.82)	0.21 (0.00, 0.67)	−0.17 (−0.70, −0.09)	0.33 (−0.12, 0.68)
Cognitive Classification	PD-NC: 3 (75.0%) PD-MCI: 1 (25.0%)	PD-NC: 4 (100.0%) PD-MCI: 0 (0%)	PD-NC: 5 (63.0%) PD-MCI: 3 (38.0%)	PD-NC: 43 (79.6%) PD-MCI: 11 (20.4%)
*Functional capacity*				
PDAQ-15	43.0 (34.5, 50.5)	51.5 (43.5, 56.5)	49.5 (48.0, 50.5)	54.0 (47.5, 57.8)
PD-CFRS	5.0 (3.5, 8.0)	2.5 (1.0, 4.0)	1.5 (0.5, 3.5)	1.0 (0.0, 3.0)
UPSA Total	81.0 (78.0, 86.5)	82.0 (79.0, 86.0)	83.0 (76.5, 86.5)	85.0 (79.0, 91.0)
Impaired/Unimpaired Baseline UPSA Total	<85: 3 (75.0%) ≥85: 1 (25.0%)	<85: 2 (50.0%) ≥85: 2 (50.0%)	<85: 6 (75.0%) ≥85: 2 (25.0%)	<85: 27 (50.0%) ≥85: 27 (50.0%)
UPSA Financial	17.0 (16.0, 19.0)	19.0 (17.0, 20.0)	18.0 (18.0, 20.0)	18.0 (18.0, 20.0)
UPSA Communication	17.0 (14.5, 17.5)	16.0 (13.5, 17.5)	15.5 (15.0, 17.0)	17.0 (13.5, 18.0)
UPSA Planning	14.0 (10.5, 18.0)	16.5 (14.5, 18.0)	16.0 (15.0, 18.0)	16.5 (14.0, 18.5)
UPSA Transportation	17.0 (16.0, 18.0)	15.5 (13.0, 19.0)	14.5 (13.0, 18.0)	16.0 (13.0, 18.0)
UPSA Household	20.0 (15.0, 20.0)	15.0 (15.0, 17.5)	17.5 (15.0, 20.0)	20.0 (15.0, 20.0)

Data presented as median (Q1, Q3) unless otherwise noted.

AES-C: Apathy Evaluation Scale, clinician reported; DRS-2: Dementia Rating Scale-2; HADS-A: Hospital Anxiety and Depression Scale, anxiety subscale; HADS-D: Hospital Anxiety and Depression Scale, depression subscale; LEDD: levodopa equivalent daily dosing; MoCA: Montreal Cognitive Assessment; ND: ‘not determined’; PDAQ-15: Penn Parkinson’s Daily Activities Questionnaire-15; PD-CFRS: Parkinson’s disease – Cognitive Functional Rating Scale; PDD: Parkinson’s disease dementia; PD-MCI: Parkinson’s disease mild cognitive impairment; PD-NC: Parkinson’s disease normal cognition; PDQ-39: Parkinson’s Disease Questionnaire; PIGD-D: postural instability and gait difficulty dominant; TR-D: tremor dominant; UPDRS: Unified Parkinson Disease Rating Scale; UPSA: UCSD Performance-Based Skills Assessment.

Regarding PD disease characteristics, the DD group had the most severe UPDRS Part III score followed by FDCM with the functional maintainer groups (CDFM/DM) the least severe, but the CDFM group had higher LEDD. PIGD-D was the predominant motor classification for each of the longitudinal trajectory groups; however, the functional decliner groups (DD/FDCM) had more participants with postural instability than the functional maintainer groups (CDFM/DM). The FDCM group had a higher PDQ-39 suggesting lower quality of life.

From a functional assessment standpoint, PDAQ-15 was lower and PD-CFRS was higher in the DD group. The UPSA Planning subscore was lower in the DD group, but there are no clear differences in UPSA total score between groups; however, the functional decliner groups (DD/FDCM) had a higher percentage of participants with baseline impaired UPSA total scores (75.0%) compared to the non-functional decliner groups (CDFM/DM; 50.0%).

### 3.5. Case descriptions of variable functional and cognitive trajectories

Table 5 displays longitudinal demographic and clinical features of select case examples. Notable commonalities between the cases include similar PD duration (range 5–7 years), baseline MoCA scores within unimpaired range (≥ 26), PD-NC cognitive classification maintained throughout study participation, equal baseline and largely stable Lawton iADL, and DRS-2 scores all above previously proposed ≤ 123 (Llebaria et al., 2008) and ≤ 133 (Turner and Hinson, 2013) cutoffs for dementia in PD.

**Table 5:** Case examples of heterogeneous cognitive and functional trajectories.

	Cognitive and functional decliner (DD)	Cognitive decliner, functional maintainer (CDFM)	Functional decliner, cognitive maintainer (FDCM)	Cognitive and functional maintainer (DM)
Study visits (longest follow-up duration, days)	BL, Y4 (1516)	BL, Y2, Y4 (1450)	BL, Y1, Y3 (980)	BL, Y1, Y4 (1397)
*Demographic characteristics*				
Age (years)	66 (70)	61 (65)	65 (68)	69 (72)
Sex	Male	Female	Male	Female
Education (year)	16	16	16	18
*Disease characteristics*				
PD duration (years)	6 (10)	5 (8)	6 (7)	7 (10)
UPDRS Part III (total)	37 (25)	11 (44)	17 (19)	23 (27)
Motor phenotype	PIGD-D (PIGD-D)	PIGD-D (PIGD-D)	PIGD-D (PIGD-D)	ND (PIGD-D)
Postural stability	2 (2)	0 (3)	0 (1)	0 (1)
LEDD (mg/day)	750 (1750)	750 (1000)	550 (1000)	400 (1075)
H&Y stage	2.5 (2.5)	2 (3)	1.5 (2.5)	2 (2.5)
PDQ-39	17 (21)	19 (35)	17 (20)	11 (9)
*Mood characteristics*				
HADS-A	4 (1)	7 (6)	5 (1)	1 (0)
HADS-D	1 (2)	2 (3)	3 (1)	1 (0)
AES-C	26 (26)	23 (18)	26 (22)	21 (18)
*Cognitive functioning*				
MoCA	28 (26)	30 (29)	27 (25)	30 (29)
Cognitive Composite	0.817 (0.202)	0.945 (0.172)	−0.076 (−0.262)	0.315 (−0.001)
Cognitive Classification	PD-NC (PD-NC)	PD-NC (PD-NC)	PD-NC (PD-NC)	PD-NC (PD-NC)
*Functional capacity*				
Lawton iADL	8 (8)	8 (7)	8 (8)	8 (8)
PDAQ-15	56 (48)	57 (52)	50 (50)	58 (57)
PD-CFRS	2 (3)	2 (3)	1 (1)	2 (0)
DRS-2	142 (135)	139 (138)	139 (140)	144 (142)
UPSA total	91 (78)	85 (82)	84 (77)	94 (95)

Data presented as baseline (longest follow-up).

AES-C: Apathy Evaluation Scale, clinician reported; BL: baseline study visit; DRS-2: Dementia Rating Scale-2; HADS-A: Hospital Anxiety and Depression Scale, anxiety subscale; HADS-D: Hospital Anxiety and Depression Scale, depression subscale; H&Y: Hoehn and Yahr scale; LEDD: levodopa equivalent daily dosing; MoCA: Montreal Cognitive Assessment; ND: ‘not determined’; PDAQ-15: Penn Parkinson’s Daily Activities Questionnaire-15; PDD: Parkinson’s disease dementia; PD-CFRS: Parkinson’s disease – Cognitive Functional Rating Scale; PD-MCI: Parkinson’s disease mild cognitive impairment; PD-NC: Parkinson’s disease normal cognition; PDQ-39: Parkinson’s Disease Questionnaire; PIGD-D: postural instability and gait difficulty; UPDRS: Unified Parkinson Disease Rating Scale; UPSA: UCSD Performance-Based Skills Assessment; Y: year 1 (Y1), year 2 (Y2), year 3 (Y3), and year 4 (Y4) study visit.

There were subtle differences between the case examples. All participant examples had similar levels of education (range 16–18 years); however, the DM participant was the only example with greater than a bachelor’s level of education. Nearly all participant examples had PIDG-D motor classification at enrollment and final follow-up, but the DM participant was the only example that started with the ND classification. Only the DD example had postural instability at baseline; however, all the participant examples had some degree of postural instability at final follow-up. All participant examples other than the DM participant reported worsening health-related quality of life (HRQL) per increasing PDAQ-39 scores (Peto et al., 1998; Margolius et al., 2018); additionally, all HADS-A/D scores were stable other than showing improvement in the FDCM example.

#### 3.5.1. Case of cognitive and functional decliner (DD)

A 66-year-old right-handed Hispanic male enrolled and participated in year 4 follow-up (Table 5). During the study period, he transitioned from an employed to unemployed status. He had improved “ON” UPDRS Part III from moderate (Higginson et al., 2013) to mild (Martínez-Martín et al., 2015; Devanand et al., 2017) with increasing LEDD and stable H&Y disease severity. His UPSA was initially in the unimpaired range (Pedersen et al., 2017) and became impaired (78) at final follow-up. His Lawton iADL was stable and PD-CFRS without a significant worsening; however, PDAQ-15 was consistent with functional worsening while maintained within the non-demented range (Brennan et al., 2016) and DRS-2 score approached the more recently suggested cutoff (≤ 133) for dementia (Turner and Hinson, 2013).

#### 3.5.2. Case of cognitive decliner/functional maintainer (CDFM)

A 61-year-old right-handed White female participated in year 2 and 3 follow-ups (Table 5). She was retired throughout her participation. Her UPDRS Part III scale progressed from mild (Anang et al., 2017) to moderate (Martínez-Martín et al., 2015; Sulzer et al., 2020) with increasing LEDD. Her H&Y progressed from stage 2–3. Her UPSA score was largely stable but transitioned from the cutoff for unimpaired at baseline (Urso et al., 2021) to impaired at final follow-up (Goldman et al., 2013). She lost one point for Lawton iADL and worsening score for PDAQ-15 but still within the non-demented range (Brennan et al., 2016), but without a significant increase in PD-CFRS (Kulisevsky et al., 2013).

#### 3.5.3. Case of functional decliner/cognitive maintainer (FDCM)

A 65-year-old right-handed White male participated in year 1 and 3 follow-ups (Table 5). He was employed at enrollment and retired during his participation. He had stable mild motor symptoms in the setting of increasing LEDD and worsening H&Y from stage 1.5 to 2.5. His baseline MoCA score was unimpaired (Bruderer-Hofstetter et al., 2020) and follow-up score impaired (Devanand et al., 2017) while maintaining cognitive function per formal neuropsychological assessment. Despite declining UPSA total score that was impaired at baseline (Wojtala et al., 2019) and final follow-up (Burnham et al., 2015), other measures of functional status were all stable (e.g., Lawton iADL, PDAQ-15, PD-CFRS, and DRS-2).

#### 3.5.4. Case of cognitive and functional maintainer (DM)

A 69-year-old right-handed White female participated in year 1 and 4 follow-ups (Table 5). She was retired throughout her participation. She had stably mild motor severity (Martínez-Martín et al., 2015) with increasing LEDD and slightly increased H&Y from stage 2 to 2.5. In addition to UPSA, all other functional assessments (i.e., Lawton iADL, PDAQ-15, PD-CFRS, DRS-2) were stable.

## 4. Discussion

Our group previously demonstrated that UPSA performance can discriminate cognitive classifications in PD cross-sectionally (Holden et al., 2018). The primary goal of this study was to validate the use of UPSA as a longitudinal PBFA in PD with the broader goal of being able to use this scale as an outcome measure in treatment trials for PD cognition and related daily function. Delineation of UPSA scores over time among a heterogenous group of people with PD will help define clinically meaningful changes for treatment effects. Longitudinal UPSA total scores were without floor or ceiling effects, similar to our validation study (Holden et al., 2018), therefore supporting the use of UPSA in longitudinal studies.

A secondary goal was to determine whether the UPSA may identify individuals with PD at highest risk of developing future cognitive decline. Our results reported here are early interim observations. UPSA and CCS remained significantly correlated at each timepoint, excepting year 3 possibly due to the smaller number of participants for this timepoint. With linear prediction modeling, baseline UPSA total score predicted CCS for each follow-up timepoint for both baseline impaired and unimpaired UPSA groups. However, baseline UPSA did not predict the rate of change in CCS over time, and therefore did not identify those individuals at greatest risk of cognitive decline in our cohort.

In the only other published longitudinal PBFA study in PD, participants with or without cognitive impairment repeatedly performed the Multiple Object Test (MOT) to determine its predictive value (Beyle et al., 2018). Participants with cognitive impairment had increased MOT omission errors over time compared to participants without (Beyle et al., 2018). Baseline omission errors were unable to predict conversion from cognitively intact to cognitively impaired, but an increase in MOT omission errors was associated with new onset PDD at follow-up (Beyle et al., 2018). The mean duration of follow-up for the MOT study was on par with the duration of follow-up for our study, therefore the absence of predictive value for each may be due to limitations in capturing cognitive decline in a PD population over a relatively short period of time.

Similar to Beyle et al. (2018) cohort, our cohort displayed heterogeneous longitudinal cognitive trajectories. Some participants with PD-MCI at baseline were stable over time, but others reverted to PD-NC or progressed to PDD. Of note, individuals with PD-MCI that revert to PD-NC still have an increased risk of later converting to PD-MCI and PDD when followed for an additional 4–5 years (Pedersen et al., 2017; Jones et al., 2018). We also observed heterogeneous functional trajectories determined by UPSA total score over time, which differs from Beyle et al. (2018) cohort that displayed increased total errors and processing time with longitudinal MOT assessments. Therefore, our cohort displayed heterogeneous trajectories for both formal neuropsychological assessment and PBFA.

The largest group with cognitive and/or functional decline was FDCM, which may represent motor confounding and/or early functional impairment prior to cognitive decline; as such, the longer longitudinal outcomes of the FDCM group, especially regarding cognitive decline, is of particular interest. It is also possible that additional factors other than cognition are contributing significantly to the variance in daily function (Bruderer-Hofstetter et al., 2020); therefore, baseline UPSA total score does not predict CCS rate change in isolation. The relationship between cognitive and functional abilities in PD are complex, including observations of cognitive deficits without functional impairments (Caviness et al., 2007), functional deficits without cognitive impairments (Foster and Doty, 2021), and correlation between functional and cognitive impairments (Sabbagh et al., 2007); therefore, prediction models using both cognitive and functional abilities can be difficult to interpret. Additionally, given the low conversion rate to PDD during this longitudinal study, predicting the risk of developing PD dementia was not possible.

There are patterns that may be helpful to understand the heterogeneous trajectories for cognition and function despite the small sample sizes. The functional maintainer groups (CDFM/DM) were younger than the functional decliner groups (DD/FDCM), highlighting that other variables in addition to neurodegenerative disease characteristics likely contribute to functional ability (Bruderer-Hofstetter et al., 2020). The DD group had longer disease duration (Hely et al., 2008), worse motor scores (Schrag et al., 2017), and more apathy (Santangelo et al., 2015; Martin et al., 2020), which have all been correlated with cognitive decline. Motor classification was primarily PIGD-D across all groups. PIGD-D is associated with PDD (Wojtala et al., 2019); however, it has also been suggested that postural instability, not PIGD-D subtype, is predictive of cognitive decline (Urso et al., 2021). Despite this, postural instability was more common in the functional decliner groups (DD/FDCM) compared to the functional maintainer groups (CDFM/DM) rather than delineated by cognitive trajectory status. The FDCM group had worse HRQL, perhaps due to declining functional abilities in the setting of maintained cognitive function and therefore associated maintained insight.

For the functional decliner groups (DD/FDCM), self-reported functional assessments provided variable results; specifically, the median PDAQ-15 was lower, but only approaching cut-off for dementia in the DD group (Brennan et al., 2016). Conversely, the PD-CFRS appeared to be more sensitive to cognitive decline than functional decline with higher scores for the cognitive decliner groups (DD/CDFM) compared to the cognitive maintainer groups (FDCM/DM; Kulisevsky et al., 2013). This highlights the importance of understanding the limitations of self-reported functional scales as outcome measures in clinical trials, since not all self-reported measures may capture functional limitations. Baseline UPSA total scores were also not clearly associated with longitudinal functional decline, such that the functional decliners (DD/FDCM) do not have lower baseline UPSA total score compared to the functional maintainers (DM/CDFM); however, the functional decliner groups (DD/FDCM) had a greater proportion of participants with baseline impaired UPSA total scores compared to the functional maintainer groups (CDFM/DM). It is unclear whether there are different overall functional trajectories or simply different timepoints along a similar functional trajectory.

Given the heterogeneity of both cognitive and functional trajectories in our cohort, we attempted to gain further insight through case examples. There were multiple commonalities in cases between the groups, including PD duration, level of education, unimpaired baseline MoCA scores, and maintained PD-NC classification. Most of the participant examples were retired or became retired during the study except the DD example newly became unemployed. All examples reported worsening health-related quality of life other than the DM example. None of the participants met the PDAQ-15 (Brennan et al., 2016) or higher DRS-2 (Turner and Hinson, 2013) cut-offs for dementia, but notably only the cognitive decliners (DD/CDFM) met the PD-CFRS cut-off for functional impairment (Kulisevsky et al., 2013) again suggesting this measurement may be more sensitive to cognitive rather than functional decline. All the decliners regardless of cognitive or functional designation (DD/CDFM/FDCM) had a decline in UPSA score at final follow-up, whereas the DM example had a stable UPSA score.

There are limitations to our study. First, the longitudinal repeated measures are the result of a convenience sample; all participants without PDD were invited for repeat visits, but those available and willing to participate could be affected by variables of interest in our study (e.g., worsening health status, independence in daily function). Of note, participants with repeat visits had statistically higher baseline MoCA scores and CCS compared to participants with a single baseline visit. Additionally, not following participants with PDD at baseline longitudinally may have skewed our data towards participants with higher cognitive functioning either with or without functional decline; this may have also limited the validity of our model with increasing years of participation. Second, our longitudinal study occurred in the setting of the COVID-19 pandemic, which may have further biased our sample. Thirdly, statistical analysis could not be performed between cognitive and functional trajectory groups given small numbers in most groups, therefore requiring us to depend on descriptive approaches. Lastly, the follow-up is relatively short for this highly educated cohort. In a cohort with a similar level of education (mean 16 years), approximately half of individuals with PD-NC developed cognitive impairment within 6 years and all new cases of PD-MCI progressed to PDD within 5 years (Pigott et al., 2015), therefore 3 years of follow-up will not capture all cases of progression to a more advanced cognitive class. As such, to capture even subtle decline, > 0.5 SD cutoff was chosen for both UPSA and CCS. A cutoff of > 0.5 SD decline in neuropsychological score is not without precedent to identify subtle cognitive decline (SCD; Zanchi et al., 2017). However, there is a move towards operationalized definitions of SCD in Alzheimer’s (Edmonds et al., 2015; Thomas et al., 2018) and PD (Jones et al., 2021) research using alternative neuropsychological measures. Notably, there is not yet a consensus on defining objective SCD (Obj-SCD). Using other prior definitions of Obj-SCD to predict cognitive decline to PD-MCI or PDD in this cohort could be of interest in the future. Clearly, additional follow-up for up to at least 6 years will capture even more cognitive decline.

To our knowledge, this study is only the second validation of a longitudinal PBFA specifically in PD (Beyle et al., 2018). Given the importance of assessing functional abilities in addition to formal neuropsychological testing in neurodegenerative disorders, our study adds to the literature on longitudinal PBFA specifically in PD-associated cognitive impairment. Given the current small body of literature on the longitudinal use of PBFAs in PD, our study is a first step. It is important to understand the utility of PBFAs for longitudinal studies interested in assessing functional impairments as well as to determine whether subtle changes in iADLs may predict future dementia. Given the heterogeneity of cognitive and functional trajectories, it will be imperative to follow larger PD cohorts for longer durations.

Although not included in the current analysis, a future goal is to determine whether specific cognitive domains are associated with UPSA performance longitudinally. Although formal neuropsychological testing can offer insight into possible impacts on iADLs, PBFAs may offer additional, complementary information including in milder forms of functional difficulties (Giovannetti et al., 2021). Therefore, the inclusion of a comprehensive neuropsychological battery allows for investigation of associations between PBFA and “pure” cognitive testing over time. Similar to our study, a comprehensive cognitive battery was performed alongside the MOT by Beyle et al. (2018), allowing for the analysis that worsening quantitative MOT score was associated with decline in attention/executive function and visuo-constructive domains. It will be informative in the future to determine whether decline in specific neuropsychological domains is also associated with decline in UPSA total scores. Similarly, it would be interesting to explore whether performance on specific neuropsychological domains is associated with specific UPSA subdomain scores.

Another future area of interest is the potential for sex-differences in UPSA performance specifically in a PD population. Although not significant, there was a greater proportion of women participants with unimpaired baseline UPSA total scores compared to impaired. Additionally, the DM group had a higher proportion of women than all the other groups. In non-demented community-dwelling older adults administered UPSA and a brief version of UPSA (UPSA-B) containing only the Finance and Communication subdomains, women had statistically insignificant lower total scores (Becattini-Oliveira et al., 2019). However, in a separate study including healthy younger participants, women had slightly superior performance compared to men on UPSA-B Finance subscore (Vella et al., 2017). Therefore, determining whether there are sex-differences in UPSA performance, and more generally PBFAs used for clinical trial assessments, is important to clarify in the future.

Ultimately, the goal is to use PBFAs to better understand the impact on daily function related to cognition for people living with PD and to accurately assess for changes in cognitive functional status both for reliable and early diagnosis of cognitive impairment as well as definition of clinically meaningful treatment effects in trial settings. Furthermore, utilization of PBFA as outcome measures in clinical trials for cognitive enhancement in PD could ensure patient-centered, clinically significant outcomes. Cognitive decline in PD need not be inevitable, as suggested in trials showing improvement in cognitive function with cognitive training (Sammer et al., 2006; París et al., 2011; Edwards et al., 2013; Naismith et al., 2013; Pena et al., 2014), physical activity (Tanaka et al., 2009; Cruise et al., 2011; McKee and Hackney, 2013; David et al., 2015; Picelli et al., 2016), and multi-disciplinary approaches (Meloni et al., 2021), as well as the hope for disease-modifying pharmacological applications in the future. Having reliable tools for measuring cognitive functional improvements longitudinally, in addition to improvement in neuropsychological outcomes, is of utmost importance.

## Data availability statement

The raw data supporting the conclusions of this article will be made available by the authors, without undue reservation.

## Ethics statement

The studies involving human participants were reviewed and approved by Colorado Multiple Institutional Review Board. The patients/participants provided their written informed consent to participate in this study.

## Author contributions

TC was involved in the review/critique of the statistical analysis, writing of the first draft of the manuscript, review/critique of subsequent manuscript drafts, and completion of the final manuscript. AF and KO were involved in the conception of the research project, execution of the statistical analysis, and review/critique of the manuscript. NL-E and AS were involved in the research project execution. LM was involved in the conception and execution of the research project, review/critique of the statistical analysis, and review/critique of the manuscript. SH was involved in the conception, organization, and execution of the research project, design and review/critique of the statistical analysis, and review/critique of the manuscript. All authors contributed to the article and approved the submitted version.

## Funding

This work was supported by the Michael J. Fox Foundation for Parkinson’s Research (Grant No.: 10879), NIH/NCATS Colorado CTSI (Grant No.: UL1TR002535), and NIH/NINDS Loan Repayment Program Award (Grant No.: L30 NS103315). Its contents are the authors’ sole responsibility and do not necessarily represent official NIH views.

REDCap services possible through the Colorado Clinical and Translational Sciences Institute Development and Informatics Service Center (DISC) grant support (NIH/NCRR Colorado CTSI Grant Number UL1 RR025780).

## Conflict of interest

The authors declare that the research was conducted in the absence of any commercial or financial relationships that could be construed as a potential conflict of interest.

## Publisher’s note

All claims expressed in this article are solely those of the authors and do not necessarily represent those of their affiliated organizations, or those of the publisher, the editors and the reviewers. Any product that may be evaluated in this article, or claim that may be made by its manufacturer, is not guaranteed or endorsed by the publisher.
